# The impact of spiritual care on the psychological health and quality of life of adults with heart failure: a systematic review of randomized trials

**DOI:** 10.3389/fmed.2024.1334920

**Published:** 2024-04-17

**Authors:** Guangwei Zhang, Qiyu Zhang, Fan Li

**Affiliations:** ^1^School of Nursing, Jilin University, Changchun, China; ^2^The First Hospital of Jilin University, Changchun, China; ^3^Department of Pathogenobiology, The Key Laboratory of Zoonosis, Chinese, Ministry of Education, College of Basic Medicine, Jilin University, Changchun, China; ^4^The Key Laboratory for Bionics Engineering, Ministry of Education, Jilin University, Changchun, China; ^5^Engineering Research Center for Medical Biomaterials of Jilin Province, Jilin University, Changchun, China; ^6^Key Laboratory for Health Biomedical Materials of Jilin Province, Jilin University, Changchun, China; ^7^State Key Laboratory of Pathogenesis, Prevention and Treatment of High Incidence Diseases in Central Asia, Urumqi, Xinjiang, China

**Keywords:** heart failure, spiritual care, psychological health, quality of life, randomized controlled study

## Abstract

**Background:**

Heart failure (HF) brings not only physical pain but also psychological distress. This systematic review investigated the influence of spiritual care on the psychological well-being and quality of life in adults with HF.

**Methods:**

We conducted a systematic literature review following PRISMA guidelines, searching seven electronic databases for relevant randomized controlled studies without language or temporal restrictions. The studies were assessed for quality using the Cochrane Bias Risk tool.

**Results:**

A total of 13 studies (882 participants) were reviewed, investigating interventions such as religion, meditation, mental health, cognitive interventions, and spiritual support. Key factors influencing the effectiveness of spiritual care implementation included integration into routine care, respect for diversity, patient engagement, intervention quality, and alignment with patient beliefs. The majority of the studies indicated that spiritual care has a potentially beneficial impact on the mental health and quality of life of patients with HF.

**Conclusion:**

The findings provide valuable insights for healthcare professionals, highlighting the importance of adopting a spiritual care approach to healthcare for this population.

## Introduction

HF is a prevalent and debilitating chronic disease (1), affecting millions of individuals globally (2). It is a complex syndrome characterized by the heart’s inability to pump sufficient blood to meet the body’s metabolic demands (3, 4), resulting in symptoms such as fatigue, dyspnea, and fluid retention (5). As HF progresses, individuals may experience physical limitations, reduced exercise tolerance, and recurrent hospitalizations, significantly impacting their overall functional capacity and independence (6, 7). The psychological burden is significant, with patients experiencing anxiety, depression, fear of worsening symptoms, and uncertainty about the future (8, 9). Managing HF involves multifaceted medical treatments, lifestyle modifications, and psychosocial support from professionals and family (10). Despite medical advancements, HF still carries high morbidity, diminished quality of life, and increased mortality rates (11, 12). The physical and psychological challenges of HF require comprehensive, patient-centered care (13). As the global population ages, the prevalence of HF is expected to increase significantly (14), highlighting the importance of exploring innovative interventions to improve adults with this condition (15). In recent years, healthcare professionals and researchers have increasingly recognized the importance of addressing psychological and spiritual aspects of health in addition to traditional medical approaches (16). Spiritual care, defined as providing support in individuals’ search for meaning, purpose, and connection with something greater than themselves (17, 18), has emerged as a potential complement to conventional medical management for chronic diseases, including HF (19).

Spiritual care recognizes the importance of psychological, emotional, and spiritual factors in holistic well-being and coping (20). Beyond religious aspects, it includes practices like prayer, meditation, mindfulness, counseling, and social support (21). In healthcare settings, spiritual care aims to address existential concerns, promote resilience, instill hope and purpose, and aid in coping with chronic diseases (22). Although studies have explored spiritual care’s impact on health conditions, its influence on the psychological well-being and quality of life in adults with HF is an ongoing area of investigation (23). Recognizing the potential benefits of spiritual care is crucial as it offers a patient-centered addition to the current management of HF. However, evidence on the effectiveness of spiritual care interventions in this population is fragmented, with varying results across studies.

To address the gap in understanding the effectiveness of spiritual care in adults with HF and provide a comprehensive understanding of the topic, we conducted a systematic review. The objective of this systematic review was to determine the impact of spiritual care interventions on the psychological health and quality of life of adults with HF. Specifically, we aim to address the following research questions:

a) What are the different types of spiritual care interventions applied in the management of adults with HF?
b) What are the effects of spiritual care interventions on the quality of life and subjective well-being of adults with HF?
c) What factors influence the effectiveness of spiritual care for patients with HF?

Through this systematic review, we aimed to provide healthcare professionals, policymakers, and researchers with a comprehensive overview of the current evidence on the role of spiritual care in the management of HF. The findings may serve as a foundation for future research, leading to tailored interventions for adults with HF.

## Methods

### Design

Systematic review according to the recommendations of the PRISMA guidelines. Meta-analysis was not performed due to the inclusion of diverse interventions in the studies. Studies included in this review must be controlled trials (CTS) using a rigorous study design.

### Identification

Electronic databases were used to search the relevant literature, such as PubMed, Web of Science, Google Scholar, Science Direct, Elsevier, Springer Link, and Wiley. The initial step involved identifying keywords and phrases relevant to the research topic: “spiritual care,” “heart failure,” “adults,” and related terms. Search strategies were created by combining the identified keywords using Boolean operators (AND, OR). Search strategies: (“spiritual care” OR “spirituality” OR “spiritual intervention” OR “prayer” OR “meditation” OR “mindfulness practices” OR “religious rituals” OR “counseling” OR “support groups) AND (“heart failure”).

### Screening

Endnote 20 software was used for literature screening (24). Initially, 1459 articles were retrieved from databases. After identifying and removing 66 duplicate articles using the software, 1393 articles remained. Subsequently, the titles and abstracts of the retrieved articles were assessed, resulting in the exclusion of 1289 articles that were irrelevant to the research topic. Next, a full-text evaluation was performed on the remaining 104 articles, excluding those that did not meet the research objectives and screening criteria.

Inclusion criteria: (1) Studies that investigated the impact of spiritual care interventions on adults with HF. (2) Spiritual care interventions could encompass a variety of approaches, such as prayer, meditation, mindfulness practices, religious rituals, counseling, support groups, and any interventions that explicitly address the spiritual well-being of the participants. (3) Controlled trial study. Eligible comparators could include standard medical care, usual care without a spiritual care component, placebo, or alternative psychosocial interventions. (4) The primary outcomes of interest were related to the psychological health and quality of life of adults with HF. Psychological health outcomes could include but were not limited to measures of depression, anxiety, stress, coping strategies, and existential well-being. Quality of life outcomes could include both disease-specific measures related to HF and generic measures assessing overall well-being, physical functioning, social functioning, and emotional well-being. (5) Studies published in the English language. (6) There were no restrictions on the date.

Exclusion criteria: (1) Review articles, editorials, conference abstracts, case reports, letters to the editor, commentaries, and opinion pieces. (2) Studies that primarily involved pediatric populations or individuals with other chronic diseases unrelated to HF. (3) Studies that did not evaluate spiritual care interventions or did not explicitly address the spiritual well-being of participants. (4) Studies that did not report relevant psychological health or quality of life outcomes. (5) Studies published in languages other than English.

The two authors (nurses) screened the titles of the retrieved articles and separated potentially relevant articles. The eligibility of the relevant abstracts was reviewed independently by the two authors, who examined the abstract of each article. The two authors used standard pretest selection forms independently to assess eligibility. When necessary, other researchers would be involved to reach a consensus.

### Eligibility

**FIGURE 1:**
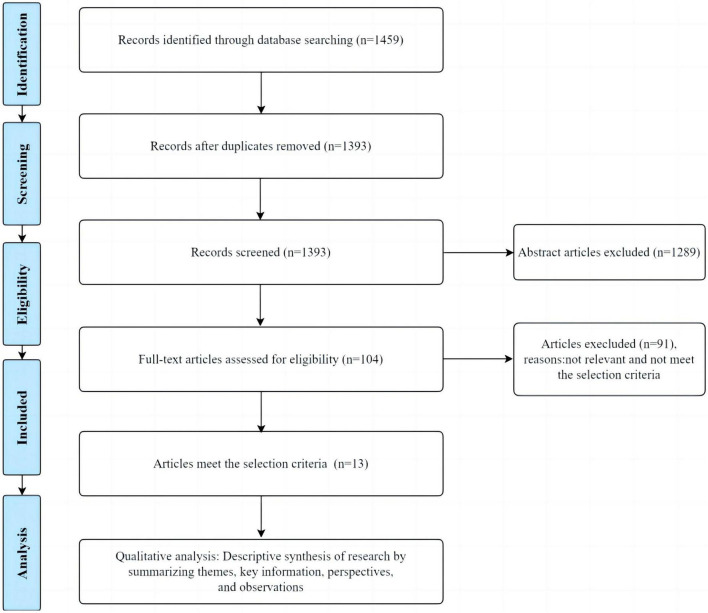
Study flowchart.

The 104 articles that met the initial screening requirements were analyzed in detail according to predefined inclusion criteria, of which 13 articles cared for adults with HF by either a spiritual or spiritual care intervention or by conventional care. At least one aspect of mental health or quality of life was measured using a questionnaire and other measurement tools (Figure 1).

### Included articles

Following a full-text evaluation of the research objectives and selection criteria, we identified relevant literature for further analysis. These selected articles closely aligned with the research objectives in theme, sample, methods, and results. The eligible literature was utilized for data extraction, analysis, and synthesis to support research conclusions and findings. Table 1 lists the relevant literature meeting the research objectives and selection criteria.

**TABLE 1:** Main characteristics of the included studies.

References	Country	Purposes	Conclusion	Limitations
Abdi et al. (29)	Ilam-Iran.	To determine the effect of religious intervention on life satisfaction and depression of the older adults.	After provision of spiritual-religious care, life satisfaction increased in the older adults with HF and their depression rate decreased. It is recommended that the nurses implement these interventions to improve the health status of the older adults.	Small homogeneous sample.
Cajanding (27)	Filipino.	To determine the effectiveness of a nurse-led cognitive behavioral intervention program on the quality of life, self-esteem and mood among Filipino patients with HF.	Nurse-led cognitive behavioral intervention is an effective strategy in improving the quality of life, self-esteem and mood among Filipino patients living with HF. It is recommended that this intervention be incorporated in the optimal care of patients with this cardiac condition.	Participants from one location only; Possible to miss long-term intervention effects; Few clinical and psychosocial variables assessed.
Creber et al. (33)	USA	To test the efficacy of a tailored motivational interviewing (MI) intervention versus usual care for improving HF self-care behaviors, physical HF symptoms and quality of life.	Patients who received the MI intervention had significant and clinically meaningful improvements in HF self-care maintenance over 90 days that exceeded that of usual care.	The loss of participants to follow-up and specifically the difference in attrition for the self-reported outcomes between the usual care and MI group.
Movahedi moghadam et al. (30)	Iran.	To determine the effect of a spiritual care program on the resilience of patients with HF.	A spiritual care program has a great role in improving the total resilience score and can be considered as a part of the holistic treatment program.	this study could not hold educational meetings in verbal way because of stress and high mortality of COVID-19, which was beyond the control of the researcher.
Curiati et al. (32)	Brazil.	To test whether meditation can reduce sympathetic activation, evaluated by norepinephrine blood levels (NE), and improve quality of life in older persons with CHF.	In older patients with optimally treated CHF, meditation reduced NE, improved quality of life, and reduced the VE/VCO2 slope.	The number of patients evaluated was small; The study did not include data about the prognosis of the patients. This study did not define the exact mechanism of action of meditation over the sympathetic system.
Binaei et al. (28)	Iran.	To determine the efficacy of hope-promoting interventions based on religious beliefs on the QOL of patients with congestive HF (CHF).	Hope-promoting intervention based on religious beliefs is a useful method for improving QOL in patients with CHF.	The sample size was small.
Steinhauser et al. (34)	USA.	To compare the efficacy of two interventions addressing emotional and existential well-being in early life-limiting illness.	Identifying appropriate measures of existential distress and growth, beyond anxiety and depression, is crucial for advances in our ability to adequately assess the mechanisms that decrease existential suffering.	Not screening for distress limits understanding for Outlook’s efficacy among palliative care patients; Low average distress levels hinder insights into Outlook’s effectiveness for highly distressed patients; Short-term follow-up (1 and 3 weeks) doesn’t capture longer-term impacts on patients and families.
Chang et al. (26)	USA.	To evaluate the efficacy of an relaxation response (RR) intervention program on the QOL and exercise capacity of CHF patients.	A short RR intervention can improve some aspects of QOL in CHF patients.	All veterans, nearly all men and with moderate CHF severity. The results are thus not generalizable to nonveterans, women, or patients with mild or severe CHF.
Jayadevappa et al. (31)	USA.	To evaluate the effectiveness of a Transcendental Meditation (TM) stress reduction program for African Americans with congestive heart failure (CHF).	Results indicate that TM can be effective in improving the quality of life and functional capacity of African American CHF patients.	First, some of the comparisons should be interpreted with caution because of the small sample size. Second, our results is limited. Last, Given the short followup, long-term effects of the intervention remain to be investigated.
Miles et al. (35)	Britain.	To test the feasibility of an RCT of a holistic care model, inclusive of spiritual support, for patients with advanced heart failure (AHF).	Overall, the key message of this study is that researchers must evaluate whether the cost of running a well-designed trial of this nature is justified in the current economic climate, where funding bodies are looking for value for money.	Fewer patients were recruited than anticipated, and recruitment and data collection took much longer than expected.
Hooker et al. (36)	America.	To evaluate the feasibility, acceptability, and preliminary evidence regarding the efficacy of a resource-sparing psychospiritual intervention to improve QOL in patients with HF.	A module-based program integrating spirituality and psychosocial coping strategies was feasible and acceptable and may improve QOL.	Pilot study in 1 region, homogenous group. Overrepresentation of male HF patients. Small sample, no control group. Missing 12-week data may impact results. Phone calls added social element, possibly affecting QOL.
Tadwalkar et al. (37)	America.	To ascertain the beneficial role of spiritual counseling in patients with CHF.	The addition of spiritual counseling to standard medical management for patients with CHF patients appears to have a positive impact on QoL.	1. Limited sample size. 2. Varying degrees of psychiatric and non-psychiatric comorbidities may impact participants’ emotional state. 3. Varying lengths of time spent during patient counseling. 4. Medication treatment.
Chang et al. (38)	Taiwan of China	To examine the effects of a tailored educational supportive care programme on sleep disturbance and psychological distress in patients with HF.	This study confirmed that a supportive nursing care programme could effectively improve sleep quality and psychological distress in patients with HF.	Single-center study; Supportive care is provided by clinical nurses only; Low prevalence.

### Data items

Data extracted from included studies were (1) author, (2) year of publication, (3) country, (4) purposes, (5) study design, (6) sample size, (7) participants, (8) intervention measures, (9) measurement methods, (10) findings, (11) conclusion, and (12) limitations. Where information was missing or unclear, we tried to contact the trial authors for further information.

### Cochrane bias risk assessment

The Cochrane bias risk assessment tool was evaluated on seven criteria, including random sequence generation, allocation concealment, blinding of participants and personnel, blinding of outcome assessment, incomplete outcome data, selective reporting, and other biases. For each item, low bias, uncertainty of bias risk, and high bias were used to judge and classify the study quality (25). The two nurses worked independently, and any discrepancies were resolved through consultation with two senior researchers.

### Analysis

Due to the heterogeneity of the studies, statistical analysis was not conducted. Instead, thematic analysis and narrative synthesis were employed to depict the study’s content. A descriptive summary of the included studies was used to organize intervention measures, participants, and outcome indicators. The characteristics of the study interventions were compared with each integrated program group. The impact of spiritual care on the quality of life and mental health in adults with HF was reviewed narratively.

## Results

### Overview of included studies

The 13 studies included were published between 2005 and 2022. The general characteristics of the selected studies are shown in Table 1. Table 2 provides a comprehensive overview of the key characteristics of the included studies in this systematic review, in study design, patient populations, interventions, and assessment outcomes. The characteristics of the included studies are summarized as follows: (1) Participants: A total of 882 patients were included in 13 studies. Diagnoses included HF (38.46%) and congestive HF (61.54%). (2) Study Group interventions: In addition to routine care, the study group included four spiritual interventions: Religion (*n* = 3), meditation (*n* = 4), mental health and cognitive interventions (*n* = 3), and spiritual support (*n* = 3). (3) Control group interventions: Most control groups received traditional usual care from their respective care providers, characterized by medical/drug-optimized therapy and HF-specific preventable risk factor modification strategies prescribed by cardiologists following Heart Association clinical practice guidelines. (4) Outcome measures: In selected studies, assessments were made on a variety of scales: Life Satisfaction and Well-being: Life Satisfaction Questionnaire (LSI-Z), Beck Depression Inventory (BDI) and QoL Enjoyment and Satisfaction (Q-LES-Q-SF); Heart Failure-Specific Quality of Life: Minnesota Living with Heart Failure Questionnaire (MLHFQ), Kansas City Cardiomyopathy Questionnaire (KCCQ) and Minnesota Living with Heart Failure Questionnaire (MLWHFQ); Self-Care and Heart Failure Management: Self-Care of Heart Failure Index (SCHFI v.6.2) and Heart Failure Somatic Perception Scale (HFSPS); Spirituality and Well-being: Connor-Davidson Resilience Scale, Parsian and Dunning Spirituality Questionnaire and Peace-Spiritual Scales; Psychological Health and Emotional Well-being: Rosenberg Self-Esteem Scale (RSES), Quick Inventory of Depressive Symptomatology (QIDS-SR16), Hospital Anxiety and Depression Scale (HADS), Cardiac Depression Scale (CDS), Profile of Mood States (POMS), Center for Epidemiologic Studies Depression Scale (CES-D), Functional Assessment of Chronic Illness Therapy Spiritual Well-Being Scale (FACITsp) and Memorial Symptom Assessment Scale (MSAS); Heart Failure Physiology and Function: VO2 and VE/VCO2 Slope, Left Ventricular Ejection Fraction (LVEF), Left Ventricular Diastolic Dimension index (LVDDi) and Brain Natriuretic Peptide (BNP); Health-Related Quality of Life (HRQoL): Health-Related Quality of Life (HRQoL), Short Form 36 (SF-36), Chinese versions of the Pittsburgh Sleep Quality Index (PSQI), Epworth Sleepiness Scale (ESS) and Quality of Life Well being Scale-Self Administered (QWB-SA); Overall Quality of Life and General Well-being: Quality of Life Index (QLI), Quality of Life at the End of Life (QUAL-E) Overall Quality of Life and General Well-being: Quality of Life Index (QLI), Quality of Life at the end of Life (Qual-e) and Functional Assessment of Cancer Therapy - General (FACT-G).

**TABLE 2:** Interventions and outcomes.

References	Design	Sample	Participants	Intervention	Measurement	Finding
Abdi et al. (29)	A randomized controlled trial.	93 (mean age > 65).	the older adults with HF disease, the patients were randomly allocated into two experimental and control groups.	The intervention done for test group was a religion-spiritual program designed based on the Richards and Bergin model, and according to Islam and Shia regulations and conducted during six sessions, each 30–45 min.	LSI-Z and BDI.	The mean of life satisfaction of the test group was higher than that of the control group. The mean of depression of the test group was lower than that of the control group.
Cajanding (27)	A randomized controlled design.	100 (mean age 55).	Filipino patients with HF. The control or the intervention group.	Control group participants received traditional care. Intervention participants underwent a 12-week nurse-led cognitive behavioral intervention program focusing on patient education.	MLHFQ, RSES, CDS.	After the 12-week intervention period, participants in the intervention group had significant improvement in their quality of life, self-esteem and mood scores compared with those who received only standard care.
Creber et al. (33)	A randomized controlled trial.	67 (mean age 62).	Patients with chronic HF.	Immediately after discharge, those in the intervention group received a single home visit and 3–4 follow-up phone calls by a nurse over 90 days. Individuals randomized to the usual care group received care as usual from their respective care providers.	SCHFI v. 6.2, HFSPS, KCCQ.	This study reports a novel nurse-led behavioral intervention that uses MI to help patients with HF improve their self-care. Although there was no statistically significant difference in the primary outcome over 90-days, there was a clinically significant difference after adjusting for confounding factors.
Movahedi moghadam et al. (30)	A randomized clinical trial.	84 (mean age 49).	Patient with HF, and randomly divided into two groups.	For the experimental group, two educational sessions were carried out in 1 h and 30 min and then continued three times a week for 1 month in order to practice spiritual care via WhatsApp. The control group did not receive the intervention that is done for the experimental group during performing intervention.	Connor-Davidson Scale, Parsian and Dunning Spirituality Questionnaire.	Dimensions of individual competence and negative emotion tolerance of resilience increased significantly in the experimental group compared to the control group and caused a significant increase in the total resilience score of the patients.
Curiati et al. (32)	A Prospective Randomized Study.	19 (mean age 74.8).	Patients with CHF.	After 2 months of optimal treatment with carvedilol, patients were randomized into two groups. The meditation group (M) was provided an audiotape, 30 min long, to listen to at home, twice a day, for 12 weeks, plus a weekly meeting. The control group (C) just had weekly meetings.	MLWHFQ, VO2 and VE/VCO2 slope, LVEF, and LVDDi.	In older patients with optimally treated CHF, meditation reduced sympathetic overactivity, improved quality of life, and ventilatory efficiency. It is simple, inexpensive, noninvasive, and easily performed. The role of meditation as a new hope in the treatment of CHF should be investigated in future trials.
Binaei et al. (28)	A randomized clinical trial.	46 (mean age 58).	Patients with CHF were selected and randomly assigned to study and control groups.	For the study group participants and their families, 60-min sessions of hope-promoting interventions based on religious beliefs were held twice a week for 3 weeks.	QLI.	The mean overall QOL score in the area of satisfaction significantly increased in the study group, compared to the controls, immediately and 1 month after the intervention. There was also a similar difference between the two groups in the area of importance immediately and 1 month after the intervention.
Steinhauser et al. (34)	A randomized control trial.	135 (mean age 62).	Patients with CHF. Patients were randomly assigned 1:1 to Outlook intervention, and relaxation meditation(RM).	Arm 1 received the Outlook intervention, addressing issues of life completion and preparation, Arm 2 received relaxation meditation(RM).	QUAL-E, FACT-G, POMS, CES-D, and FACITsp.	Compared to RM, Outlook participants did not have significant differences over time in the secondary outcomes of overall quality of life, anxiety, depression, FACT-G subscales, and FACIT-sp subscales. qualitative results suggested that respondents found the intervention to be useful by allowing them.
Chang et al. (26)	A randomized control trial.	95 (mean age 69).	Patients with moderate severity CHF.	relaxation response (RR) group, usual care (UC) group.	Peace-spiritual scales.	The RR group had significantly better QOL change scores in peace-spiritual scales than did the UC group. No statistically significant intervention effect on physical QOL or exercise capacity was observed.
Jayadevappa et al. (31)	A randomized control Study.	23 (mean age 64.1).	African Americans with CHF. Participants were randomized to either TM or health education (HE) group.	Transcendental meditation (TM) is a behavioral intervention for stress reduction that is widely used and validated. It is practiced for 15–20 min twice daily while sitting comfortably with eyes closed. Participants in the HE program attended educational sessions that paralleled the time spent in TM training and practice. Health education (HE) participants were instructed to listen to music or read for 20 min twice a day.	HRQoL, SF-36, LHFQ, QWB-SA, PSS, CES-D, BNP.	They research yields information on the potential effectiveness of TM in improving HRQoL and physiologic outcomes for CHF in this high-risk population.
Miles et al. (35)	A randomized controlled. Feasibility Study.	47 (mean age 80)	AHF patients were randomized to control (standard care, *n* = 25) or intervention (standard care plus spiritual support, *n* = 22) groups.	Spiritual support consisted of a 1-h discussion facilitated by trained volunteers using a “Spiritual Enquiry Tool” at two-monthly intervals over 6 months.	QoL.	(i) the possible positive effect of spiritual support on QoL and anxiety, and (ii) possible lower NHS resource use and cost savings in patients receiving spiritual support.
Hooker et al. (36)	A clinical controlled trial.	66 (mean age 61.6)	Adults with HF. Multicomponent intervention (made of Education, Reflection, Activity, and Spirituality); standard care.	A 12-week mail-based intervention addressing spirituality, which have three sections: (1) educational information, addressing different psychospiritual topics, (2) questions to aid in reflecting on the material, and (3) behavioral activities eliciting practice of the new skills.	KCCQ, PHQ-9, meaning in life Questionnaire, and FACITsp.	A multicomponent intervention (made of Education, Reflection, Activity, and Spirituality) improved the quality of life compared to standard care.
Tadwalkar et al. (37)	Pilot study, Randomized controlled trial.	23 (mean age 57)	Religious (*n* = 14) and Non-religious (*n* = 9).	Religious: the chaplains, comprised of various activities related to religious counseling. Non-religious: the volunteers, comprised of personal discussions, recreational activities, and undertakings related to bolstering social and spiritual support.	QIDS-SR16, FACIT-Sp-Ex, MSAS, Q-LES-Q-SF.	An overall enhancement in QoL, as established by improved survey scores with the use of adjunct religious or spiritual support in patients admitted to a hospital with HF.
Chang et al. (38)	A randomized controlled trial.	84 (mean age 72.8)	Adults with HF. The intervention group (*n* = 43) and the control group (*n* = 41).	Patients in the intervention group received a 12-week tailored educational supportive care programme including individualized education on sleep hygiene, selfcare, emotional support through a monthly nursing visit at home, and telephone follow-up counseling every 2 weeks. The control group received routine nursing care.	PSQI, ESS, HADS.	Compared with the control group, the intervention group had significantly greater improvement in sleep quality (*p* < 0.001), daytime sleepiness *p*< 0.001), anxiety (*p* < 0.001), and depression (*p* < 0.001) after 12 weeks of the intervention.

### Quality of included studies

**FIGURE 2:**
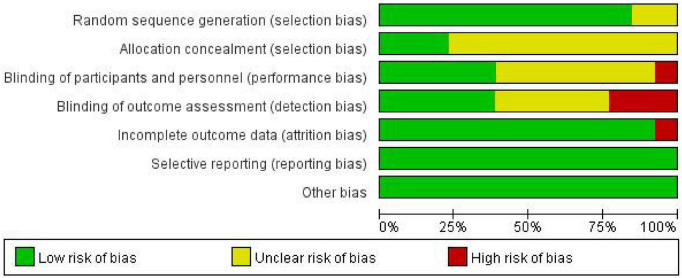
Risk of bias graph: review authors’ judgments about each risk of bias item presented as percentages across all included studies.

**FIGURE 3:**
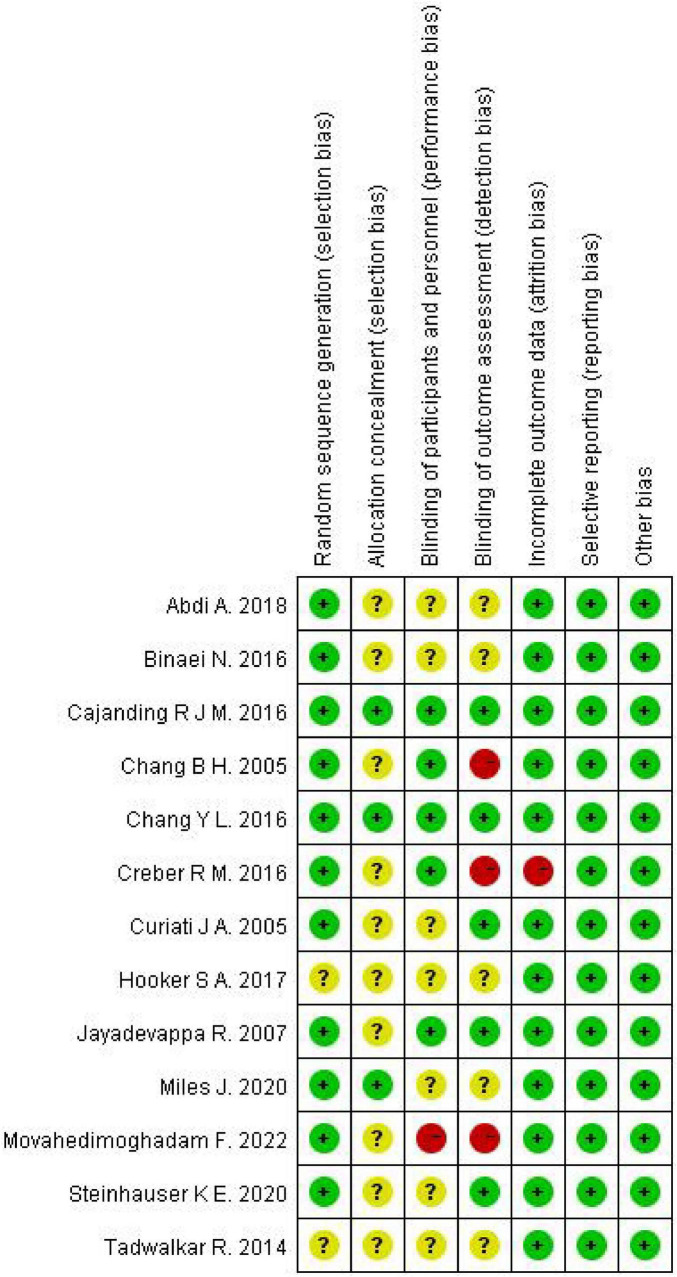
Risk of bias summary: review authors’ judgments about each risk of bias item for each included study.

The Cochrane Risk of Bias tool revealed moderate potential bias across all studies, focusing on critical domains such as random sequence generation and allocation concealment. The 13 studies (26–38) meticulously detailed the processes involved in generating the random sequence, utilizing methods such as random number tables and computer-generated randomization. However, 10 studies (26, 28–34, 36, 37) were unclear about adequate measures for concealing allocation. Given the nature of spiritual care interventions, blinding of participants was unfeasible in the majority of studies. Nevertheless, 5 studies (27, 31, 32, 34, 38) blinded outcome assessors in the intervention group. Regarding attrition bias, only one study mentioned attrition without specifying reasons (33). In contrast, other studies provided detailed explanations for participant dropouts. In summary, the moderate risk classification of included studies highlights the need for cautious interpretation and emphasizes the importance of considering potential biases while assessing the evidence. Figures 2, 3 provide detailed insights into each study’s risk of bias.

### The impact of spiritual care

A total of 13 studies (26–38) investigated the impact of spiritual care on mental health and quality of life in patients with HF. 9 of these studies (26–32, 37, 38) reported that spiritual care was associated with statistically and clinically significant improvements in mental health and quality of life. The effects of spiritual care are as follows:

(1) Quality of life and satisfaction

11 studies (26–29, 32–38) evaluated the impact of spiritual care on the quality of life of patients with HF. Among them, 8 studies involving interventions such as meditation (26, 32, 34), religious beliefs (28, 29, 37), spiritual support (38) and cognitive interventions (27) demonstrated significant improvement in the life quality of the intervention group, with mean life satisfaction higher than that of the control group. 3 studies (33, 35, 36) showed no significant difference in the quality of life between the intervention and control groups.

(2) Emotional health

8 studies (26, 27, 29, 31, 32, 34, 37, 38) assessed the impact of spiritual care on emotional health (anxiety, depression). In these 8 studies, the intervention group exhibited improvements in overall scores on scales related to emotional well-being, such as the POMS and CESD scale, compared to the control group, leading to an enhancement in patients’ emotional health.

(3) Physical function

Three studies (31–33) evaluated the impact of spiritual care on physical function. Two studies (31, 32) indicated that meditation led to improvements in physical functions, such as blood pressure levels and exercise tolerance, among the intervention group. However, one study (33) suggested that tailored Motivational Interviewing (MI) intervention showed no difference in improving physical symptoms of HF compared to standard care.

(4) Mental Health

Two studies (28, 30) reported the impact of spiritual care on mental health. The results showed a statistically significant increase in spiritual scores and improved mental well-being for the experimental group after the intervention (t_72_ = 0.66, *p* = 0.511).

## Discussion

The findings of this systematic review have significant implications for the field of spiritual care, especially for patients with HF. After reviewing the studies, it is evident that existing theories and models of spiritual care strongly support the role of spirituality in enhancing patients’ well-being. The positive outcomes observed in mental health and quality of life support the theoretical frameworks proposing a holistic approach to patient care, considering physical, psychological, and spiritual dimensions. Furthermore, the variety of interventions utilized throughout the studies presents a new perspective on how spiritual care can be customized to fulfill the unique needs of patients with different cultural backgrounds, religious beliefs, and personal values. This indicates that spiritual care is an adaptive practice that can be adapted to different theoretical foundations while respecting individual variations. In addition, the findings highlight the importance of incorporating spiritual care interventions into the clinical management of patients with HF. Integrating spiritual care into the comprehensive treatment plan can provide emotional support, improve coping mechanisms, and increase overall quality of life. Healthcare professionals should receive training and resources to communicate effectively about spiritual care. Developing educational materials and guidelines that respect patients’ cultural and religious beliefs can improve spiritual care interventions. It is also crucial to recognize that spiritual care is not limited to religious practices and encompasses personal beliefs and practices that contribute to patients’ emotional and psychological well-being.

In managing patients with HF, medical treatment, lifestyle adjustments, and emotional well-being are crucial aspects. Spiritual care is a nursing approach that prioritizes the spiritual and inner needs of patients. It emphasizes the cultivation of inner calm and hope, aiming to improve their psychological well-being and quality of life (39). The study indicated that spiritual care can improve the psychological well-being and quality of life of cancer patients and their family caregivers (40, 41). Vespa et al. (42) found that mindfulness training can enhance emotional and cognitive balance, as well as happiness, for early-stage Alzheimer’s disease patients and their caregivers, thereby improving their quality of life. Spiritual care encompasses various religious and non-religious practices, aimed at helping patients find inner strength and meaning and establishing connections with others, nature, and a higher power (43). In this study, we have identified several key factors that influence the effectiveness of spiritual care: (1) Alignment with patient beliefs and values. Tailoring spiritual care to align with a patient’s beliefs and values is essential. Studies have shown that spiritual care is more effective when it resonates with an individual’s personal belief system, ensuring the intervention is meaningful and relevant to their spiritual journey (44). (2) Quality and relevance of the intervention. The impact of spiritual care on patients is influenced by its quality, relevance, and appropriateness (31, 32, 37, 38). Interventions like meditation, when well-designed and relevant to the patient’s needs, have demonstrated improvements in various aspects of well-being, highlighting the importance of a well-crafted intervention. (3) Patient participation. Actively involving patients in spiritual care and encouraging their participation can significantly impact its effectiveness (34). Patients who actively engage in spiritual practices and discussions often experience deeper benefits, indicating the importance of patient involvement in the process. (4) Respect for diversity and cultural competence. The effectiveness of spiritual nursing interventions is significantly influenced by cultural, religious, and individual contexts. Recognizing and respecting the diversity of spiritual and religious beliefs among patients is crucial. Cultural competence and sensitivity ensure that spiritual care is tailored to individual cultural contexts, making it more effective and meaningful for the patient (45). (5) Integration into routine care. To achieve the best treatment outcomes, it is crucial for healthcare providers, to integrate spiritual care into routine healthcare practices (27, 30, 38). When healthcare providers integrate spiritual care with medical treatments, patients experience better outcomes.

With the healthcare landscape evolving, virtual mental nursing platforms offer exciting opportunities to enhance spiritual care for patients with HF (46). This adaptability to modern healthcare contexts while retaining core spiritual care principles is a testament to the dynamic nature of the field. From a clinical perspective, incorporating technology into spiritual care interventions offers promising prospects. For example, virtual spiritual care platforms can help overcome the challenges of social distancing and limited physical interactions (47). Particularly relevant in the context of pandemics and widespread digital connectivity, these platforms provide patients with a means to access spiritual care remotely, transcending barriers like transportation, physical limitations, or geographical distances (48). This technological evolution not only improves patient access to care but also highlights the adaptability of spiritual care practices to meet the demands of modern healthcare (49). Methodologically, the introduction of virtual spiritual care platforms brings new considerations. These technologies broaden the feasibility and scalability of spiritual care interventions, potentially reaching a wider patient population (50). However, it is crucial to substantiate the effectiveness and safety of these interventions through rigorous research. Comparative studies that evaluate outcomes between traditional in-person spiritual care and technology-driven interventions can establish the reliability and validity of these innovative methods. Despite these advancements, it is important to acknowledge potential limitations. Patient technological literacy, device accessibility, and comfort with virtual interactions may impact the feasibility and acceptance of technology-driven interventions (51). Ensuring inclusivity is crucial, requiring investigations into these aspects to prevent any unintended exclusion of certain patient groups (52). Additionally, the ethical considerations of utilizing virtual mental nursing platforms in spiritual care must be addressed, encompassing concerns related to data privacy, algorithm transparency, and patient autonomy (53, 54).

The methodological considerations arising from the reviewed studies offer insights into the rationality and feasibility of spiritual care interventions. The range of spiritual care interventions used in studies highlights their adaptability to meet the unique needs of patients. Additionally, the use of validated assessment tools for spiritual care, such as those focusing on depression, quality of life, and coping mechanisms, enhances the credibility and reliability of the findings. However, the limitations identified in the reviewed studies prompt considerations for future research in this field. While the positive outcomes of spiritual care interventions are promising, biases stemming from small sample sizes and single-site studies underscore the need for larger, multicenter trials. Furthermore, the cultural and religious diversity among patients necessitates more research on specific populations to determine how different belief systems and practices interact with spiritual care interventions. Exploring the impact of interventions across various cultural backgrounds and religious affiliations can offer a nuanced understanding of how spirituality influences patient outcomes. Additionally, the potential mediating role of spirituality in influencing patients’ adherence to medical treatments and lifestyle modifications warrants further investigation.

There are some limitations in this study. First, there is methodological heterogeneity in the studies included. There are some differences in study sample size, intervention methods, and assessment tools, which may affect the accuracy of the analysis. Second, most studies have focused on patients in specific regions or cultures, which limits the generalisability and generalization of the findings. Finally, The number of included studies is limited and more studies are needed to strengthen the robustness of our findings. Therefore, the current review cannot rule out possible publication bias. Nevertheless, these results have important implications for practice, policy, and future research. For example, these findings may prompt medical professionals to re-examine and improve the care they give to patients with HF. In addition, these results may guide future research and encourage further exploration of interventions related to mental health and quality of life in patients with HF.

## Conclusion

In conclusion, this systematic review highlights the positive influence of spiritual care interventions on the psychological health and quality of life of patients with HF. It emphasizes the need for tailored interventions, healthcare professional involvement, and continued research to better understand and optimize the impact of spiritual care in this patient population. The review bridges the gap between theoretical foundations and practical applications, emphasizing the adaptive nature of spiritual care that accommodates the diverse cultural and religious backgrounds of patients.

## Data availability statement

The original contributions presented in the study are included in the article/supplementary material, further inquiries can be directed to the corresponding author.

## Author contributions

GZ: Writing – original draft, Resources, Methodology, Formal analysis, Data curation, Conceptualization. QZ: Writing – original draft, Validation, Software, Resources, Formal analysis, Data curation. FL: Writing – review and editing, Methodology, Funding acquisition, Formal analysis.
